# A comparative study of risk of pneumonia and mortalities between nasogastric and jejunostomy feeding routes in surgical critically ill patients with perforated peptic ulcer

**DOI:** 10.1371/journal.pone.0219258

**Published:** 2019-07-03

**Authors:** Shih-Chi Wu, Peiling Hsieh, Yi-Wen Chen, Mei-Due Yang, Yu-Chun Wang, Han-Tsung Cheng, Chia-Wei Tzeng, Chia-Hao Hsu, Chih-Hsin Muo

**Affiliations:** 1 Graduate Institute of Clinical Medical Science, China Medical University, Taichung, Taiwan; 2 Trauma and Emergency Center, China Medical University Hospital, Taichung, Taiwan; 3 Department of Pharmacy, China Medical University Hospital, Taichung, Taiwan; 4 Department of Nursing, China Medical University Hospital, Taichung, Taiwan; 5 Department of Surgery, China Medical University Hospital, Taichung, Taiwan; 6 Management Office for Health Data, China Medical University and Hospital, Taichung, Taiwan; University of Notre Dame Australia, AUSTRALIA

## Abstract

**Introduction:**

Enteral nutrition (EN) is important in the management of critically illness. Yet, the best route (e.g. pre-pyloric or post-pyloric) for EN in critically ill patients remains to be investigated, especially in specific surgical patients group. In addition, EN could be associated with a higher risk of aspiration pneumonia. Therefore, we evaluate the effect of various EN routes in surgical critically ill perforated peptic ulcer (PPU) patients who underwent surgery and required mechanical ventilation.

**Method:**

We collected data of surgical critically ill PPU patients admitted to intensive care unit. The patients were managed with appropriate care bundle and program. To reduce the impact of surgery types, we excluded those who had received other surgical procedures and included patients that only received simple closure. Patients were classified into nasogastric and jejunostomy feeding groups. The demographics, severity scores (e.g.: APACHE II, SOFA, and POSSUM), body mass index (BMI), comorbidities, ventilator days, use of proton pump inhibitors (PPIs), pneumonia occurrence, mortality and complications were collected for analysis.

**Results:**

A total of 136 critically ill PPU patients that received surgery and mechanical ventilation were enrolled. There were 53 patients in NG group and 83 patients in FJ group. There were no differences in demographics, severity scores, BMI, comorbidities, ventilator days, use of PPIs, pneumonia occurrence, mortalities and complications between groups.

**Conclusion:**

Our study indicates that there are no differences in mortalities and pneumonia occurrence using nasogastric or feeding jejunostomy in surgical critically ill PPU patients underwent surgery. However, further studies are required.

## Introduction

Nutritional support is an important issue in the intensive care unit, and enteral nutrition (EN) is recognized as a fundamental component of the management of critically ill patients [1–2]. Although nutritional therapy plays an important role in critical care, there exists controversy in the timing and the route of administering enteral nutrition to critically ill patients. Most of the studies have focused on the relationship between nutritional support and disease severity [3–4]. It has been shown that early EN can attenuate disease severity, modulate immune response, and positively affect clinical outcomes [5]. However, there are concerns that EN feeding may be associated with a higher risk of aspiration and nosocomial pneumonia [6–8].

Though there are studies concerning the route of EN in critically ill patients, the issue of the optimal route (e.g., pre-pyloric versus post-pyloric feeding) is unresolved and the relative efficacy and safety of post-pyloric feeding compared to gastric feeding in nosocomial pneumonia remain unclear [9–11]. The European Society for Parenteral and Enteral Nutrition (E.S.P.E.N.) guidelines suggests using gastric access as a standard and implementing post-pyloric access in patients intolerant to gastric feeding due to gastroparesis, and in patients with a high risk of aspiration [12].

The American Society for Parenteral and Enteral Nutrition (A.S.P.E.N.) guidelines suggest a diversion of the feeding route by post-pyloric enteral access device placement in patients at a high risk for aspiration [13]. Furthermore, meta-analyses indicate that compared to pre-pyloric feeding, small intestinal feeding can reduce the risk of pneumonia in critically ill patients without affecting mortality, length of ICU stay or duration of mechanical ventilation [14–16].

In contrast, others consider that these meta-analyses have mixed medical and surgical populations, and more attention should be given to each specific population [17]. Because there were different subgroups of critically ill patients, we were interested in investigating the impact of feeding route in surgical critically ill patients with perforated peptic ulcer (PPU) and sepsis/septic shock who underwent surgery and required mechanical ventilation.

## Materials and methods

In this retrospective study, we collected data from surgical critically ill patients with PPU and sepsis/septic shock who were admitted to our intensive care unit. We followed the guidelines from the Surviving Sepsis campaign in the management of sepsis/septic shock [18]. We also followed the guidelines of the Infectious Diseases Society of America (IDSA) for the use of antibiotics.

To reduce the confounding effect of different surgery types for PPU, we only included patients who underwent a simple closure procedure and excluded all other types of surgery such as gastrectomy with B-II anastomosis.

The feeding route is selected based on intraoperative findings. The feeding jejunostomy is indicated when there were abdominal turbid ascites/soiling or a concern of postoperative sutured site leakage. Otherwise, a simple suture procedure and nasogastric tube is considered. The operations were performed by boarded surgeons (Wu SC, Wang YC, Cheng HT, Tzeng CW, and Hsu CH).

Open laparotomy were performed in patients with unstable hemodynamics. In addition, we use laparoscopic approach first in stable patients. Conversion from a laparoscopic approach is indicated when the perforated peptic ulcer size is more than one cm in diameter with concern for possible postoperative leakage; in the presence of deteriorating hemodynamics during laparoscopic surgery; and when severe abdominal soiling occurs or there is a high possibility of postoperative leakage.

All patients received prokinetics and intravenous proton pump inhibitors (PPIs) during their ICU stay.

Independent variables include age, gender, demographics, comorbidities, clinical characteristic, pre/postoperative shock, status of microbiosis and blood transfusion, severity score (e.g., APACHE II, SOFA, and POSSUM), serum lactate, glucose, albumin level and body mass index (BMI) were all measured.

Dependent variables include the occurrence of pneumonia, reintubation, 90-day mortality, administration/establishment enteral nutrition, total ventilator days, length of stay and days of established EN after operation were considered outcome parameters and collected for analysis.

Patients with endotracheal intubation in the intensive care unit received continuous control of tracheal cuff pressure approximately 22–24 mmHg with regular monitoring. A ventilator associated pneumonia (VAP) prevention bundle was applied to reduce the risk of pneumonia [19].

To comply with the Personal Information Protection Act, the data abstracted from the chart contained no identification of patient information. All identification information was replaced with surrogate numbers for research. This study was approved by the Research Ethics Committee at China Medical University and Hospital (CMUH106-REC3-085).

### Definition and measurement

In this study, pneumonia was identified by (1) image evidence reported by the radiologist, (2) fever and low respiratory airways symptoms, (3) microbial evidence from quantitative sputum aspirate, and (4) a decrease in PaO2/FIO2 ratio. Reintubation was defined as intubation within 48 hours of extubation due to respiratory compromise. The status of microbiosis was defined by a definitive culture result from the blood stream or ascites.

In the nasogastric group, the administration of enteral nutrients was started after surgery once gastric residual volume (GRV) decreased to less than 300 ml/day. It was delayed if there was persistent hemodynamic instability. The hemodynamic instability was defined as an episode of systolic blood pressure < 90 mm Hg or a decrease in systolic blood pressure > 40 mm Hg, or a need for the use of vasopressor to keep a mean arterial pressure >65 mm Hg.

We also held EN if there was GRV drainage of more than 300 ml/day after EN attempts, regardless of prokinetics use. In the feeding jejunostomy group, the enteral nutrient administration was started within 24 hours after surgery but was held if there was persistent hemodynamic instability.

Established EN was defined as patients able to tolerate EN 500 Kcal/day smoothly. We then gradually increased the number of calories given. If a patient failed nasogastric feeding after two attempts, we arranged for radiologically guided insertion of nasojejunal tube for nasojejunal feeding (these patients were excluded from this study).

### Type of EN administration

We used a monomeric diet during ICU stay and shifted to a polymeric diet when patients transferred out of the ICU.

### Goal of calorie supplementation and PN use

In the current study, the goal of calorie supplementation is ≤ 25 kcal/kg (actual BW)/day during the acute phase (48 hours after ICU admission), and 30 Kcal/kg (actual BW)/day during the post-acute phase (>4 d post admission) [20].

If EN cannot be established within 72–96 hours after admission, we consider the use of parenteral nutrition (PN) and follow guideline suggestions for PN prescription and discontinuation [12, 21]. In patients with identified preoperative malnutrition (e.g.: BMI <18.5 kg/m2) and intolerable to enteral feeding, we start PN within 24 hours after ICU admission.

Therefore, the energy intake were initially hypocaloric nutrition that target to 20–25 kcal/kg/day and 1.5 amino acids/kg/day, then gradually increased to 30–35 kcal/kg/day. The PN could be discontinued when EN can reach a target of 60% nutritional requirements.

Therefore, two feeding routes were used in this study: pre-pyloric nasogastric feeding (NG) and surgical feeding jejunostomy (FJ).

### Assessment of patient severity

The assessment and evaluation of perioperative and operative severity in these patients were performed using physiological severity scores, including the Acute Physiology and Chronic Health Evaluation II (APACHE II) score and the Sequential Organ Failure Assessment (SOFA) score. We also used the physiological and operative severity scores for the enumeration of mortality and morbidity (POSSUM) scoring system to evaluate the physiological status, surgical mortalities and morbidities of our surgical critically ill patients. The physiological POSSUM score includes the evaluation of physiological severity, while the operative POSSUM score include operative severity, operational procedures, total blood loss, degree and extent of peritoneal soiling, mode of surgery, etc. Therefore, the POSSUM scoring system is considered reliable for surgical audits [22–23].

We use these scores (physiological-POSSUM, operative-POSSUM, and sum of POSSUM scores) together with the conventional APACHE II and SOFA scores for the evaluation and assessment of multiple preoperative and postoperative clinical parameters in our surgical patients.

### Statistical analyses

Continuous variables including age, total ventilator days, length of stay, BMI… etc. are reported as the median and interquartile range (IQR). The differences of continuous variables between NG and FJ group were tested by Wilcoxon rank sum test because they did not fit normal distribution. Discrete variables, including gender and clinical characteristics, are expressed as counts and percentages. We use a chi-squared test to evaluate the difference of discrete variables between NG and FJ groups, and Fisher’s exact test was used when patient number less than five [24]. The logistic regression was used to assess the risk and odds ratio (OR) and 95% confidence interval (CI) for pulmonary complications (included reintubation and pneumonia), infection (included bacterial and fungus), and survival in the NG group compared to the FJ group. Moreover, adjusted logistic regression was controlled for the variables that show significance and have a significant association with outcome in Table 1 [25].

**Table 1:** Distribution of gender and comorbidities between the NG and FJ feeding groups.

	NG N = 53		FJ N = 83		Fisher’s exact / chi-square test p-value
Variables	n	%	n	%	
Gender					0.63
Female	17	32.1	30	36.1	
Male	36	67.9	53	63.9	
Intravenous PPI use	53	100.0	83	100.0	1.00
Comorbidity					
DM	16	30.2	37	44.6	0.09
Hypertension	21	39.6	35	42.2	0.77
Renal disease	10	19.9	19	22.9	0.58
Heart Lung disease	14	26.4	32	38.6	0.14
Liver cirrhosis	6	11.3	15	18.1	0.29
Vasopressor use					0.45
Pre-op only	4	7.55	3	3.61	
Post-op only	10	18.9	24	28.9	
Both pre and post -op	8	15.1	10	12.1	
Blood transfusion	24	45.3	41	49.4	0.64

NG: nasogastric, FJ: feeding jejunostomy, PPI: proton pump inhibitor

We also used linear regression to estimate the difference in total ventilator days, length of stay, Δ albumin between admission and 72–96 hr. after operation, Δ APACHE between admission and discharge, Δ GCS between admission and discharge, and days of established EN after operation in the NG group compared to the FJ group. All statistical analyses were performed using SAS statistical software version 9.4 for Windows (SAS Institute Inc., Cary, NC, USA). The significance level was set at 0.05 in two-tailed tests.

## Results

From 2008 July– 2017 January, there were 161 critically ill PPU patients who received surgery. We excluded 10 patients because they received gastrectomy operations, and another 15 patients were excluded because they received nasojejunostomy. As a result, a total of 136 patients were enrolled in this study.

Among these 136 patients, 53 were in the nasogastric group (M: F = 36:17, average age: 68.8±15.5 y/o), and 83 were in the surgical feeding jejunostomy group (M: F = 53:30, average age: 70.1±15.3 y/o).

There were 25 patients in the NG group and 74 patients in the FJ group who received an open laparotomy approach. There were 28 patients in NG group and 9 patients in FJ group who received a laparoscopic approach.

There were no differences in gender, use of intravenous PPIs, comorbidities, clinical characteristics, pre/postoperative shock incidence, and blood transfusions between groups (Table 1).

In addition, there were no differences in age, total ventilator days, BMI, severity scores (APACHE II score, SOFA and POSSUM score), and no difference in admission serum lactate, glucose and albumin level between the two groups. However, there was a longer length of hospital stay in the FJ group (median: 18 vs. 13 days; p = 0.01; Table 2).

**Table 2:** Distribution of age and baseline clinical presentation between the NG and FJ feeding groups.

	NG N = 53		FJ N = 83		Wilcoxon Rank sum test p-value
	Median	IQR	Median	IQR	
Age	72.0	26.0	72.0	26.0	0.66
BMI	21.8	3.88	21.9	5.07	0.30
SOFA score	4.00	4.00	3.00	5.00	0.23
BUN	34.0	30.5	32.0	27.0	0.80
Cr	1.41	1.19	1.50	1.56	0.25
Sugar admission	156.0	93.0	143.5	78.0	0.88
Lactate admission	26.1	45.7	27.5	42.2	0.37
Albumin admission 24 hours	2.50	0.80	2.45	0.70	0.50
Albumin level 72~96 after OP	2.60	0.60	2.65	0.60	0.51
APACHE at admission	20.0	12.0	20.0	9.00	0.52
P-POSSUM score	25.5	11.5	27.0	11.0	0.80
O-POSSUM	9.00	7.00	9.00	1.00	0.36
Total POSSUM score	38.5	13.0	36.0	14.0	0.45
GCS(admission)	10.0	5.00	10.0	2.00	0.13
Total ventilator days	6.00	10.0	7.00	15.0	0.19
Length of stay	13.0	18.0	18.0	31.0	0.01

NG: nasogastric, FJ: feeding jejunostomy, IQR: interquartile range

Table 3 shows the odds ratio for outcomes in the NG group compared to the FJ group in logistic regression. There were no differences in reintubation, occurrence of pneumonia, bacterial infection (Gram positive or negative), fungal infection, and survival between groups.

**Table 3:** Odds ratio for outcomes in NG feeding compared with FJ feeding in logistic regression.

	NG		FJ		NG vs. FJ	
	n	%	n	%	OR (95% CI)	p-value
Total number						
Pulmonary Complication†	14	26.4	22	26.5	1.23 (0.90–1.68)	0.1884
Re-intubation†	5	9.43	4	4.82	1.72 (0.97–3.03)	0.0628
Pneumonia†	10	18.9	18	21.7	1.09 (0.80–1.49)	0.5970
Infection†	20	37.7	44	53.0	0.93 (0.70–1.26)	0.6525
Bacterial infection†	18	34.0	43	51.8	0.88 (0.65–1.21)	0.4371
G(-)infection†	7	13.2	15	18.1	0.89 (0.61–1.31)	0.5580
G(+)infection†	5	9.43	9	10.8	0.95 (0.61–1.49)	0.8351
Both†	6	11.3	19	22.9	0.82 (0.54–1.24)	0.3499
Fungus infection†	5	9.43	19	22.9	0.78 (0.54–1.13)	0.1854
Survival	43	81.1	64	77.1	1.09 (0.82–1.44)	0.5769

†Adjusted for length of stay

NG: nasogastric, FJ: feeding jejunostomy, OR: odds ratio, CI: confidence interval

Regarding the outcomes of the NG group compared to the FJ group in linear regression, there were no differences in total ventilator days, Δ albumin between admission and 72–96 hr. after operation, Δ APACHE between admission and discharge, and Δ GCS between admission and discharge. However, there was a longer length of hospital stay in the FJ group, and the days of established EN after operation was longer in the NG group than in the FJ group (5.68±5.20 versus 2.65±1.40; p<0.0001; Table 4).

**Table 4:** Outcomes in NG feeding compared with FJ feeding in linear regression.

	NG			FJ			NG vs FJ		
	N	Mean	SD	N	Mean	SD	Estimated coefficient	SE	p-value
Total ventilator days	53	12.1	15.8	83	16.2	22.4	-4.12	3.54	0.2461
Length of stay	53	19.3	16.9	83	29.8	27.9	-10.5	4.27	0.0155
Δ albumin at admission and 72~96 after OP	37	0.09	0.85	66	0.17	0.90	-0.08	0.18	0.6584
Δ APACHE at admission and discharge	49	-6.53	9.09	79	-6.63	9.38	0.10	1.69	0.9517
Δ GCS at admission and discharge	49	1.71	4.06	78	2.27	4.33	-0.55	0.77	0.4727
Established EN after operation (days)	53	5.68	5.20	83	2.65	1.40	3.03	0.60	<0.0001

Δ albumin at admission and 72~96 after OP = (albumin level 72~96 after operation)-(albumin level at admission)

Δ APACHE at admission and discharge = APACHE(discharge)- APACHE(admission)

Δ GCS at admission and discharge = GCS (discharge)- GCS (admission)

NG: nasogastric, FJ: feeding jejunostomy, GCS: Glasgow coma scale, SD: standard deviation, SE, standard error

## Discussion

In this study involving surgical critically ill PPU patients, there was no difference in the occurrence of pneumonia between the nasogastric and feeding jejunostomy groups (Table 3). This result showed inconsistence from previous meta-analyses that mixed with medical and surgical patients [13–16].

There were different subgroups of critically ill patients with various characteristics, inconsistences existed among studies concerning the use of gastric or post-pyloric feeding [1, 12, 26–27]. Additionally, a large multicenter randomized controlled trial showed early nasojejunal nutrition did not increase energy delivery and did not appear to reduce the frequency of pneumonia in mechanically ventilated patients [27]. Therefore, these results may indicate, at least in part, the importance of various characteristics in different subgroups of critically ill patients

The procedure of feeding jejunostomy is indicated when there were abdominal turbid ascites/soiling or a concern of postoperative sutured site leakage, which raise a concern that patients in FJ group might be more severe than those in the NG group. To minimize this bias, the physiological and operative POSSUM score were assessed. The operative POSSUM score include several major points during operation, such as: 1. number of procedures, 2. estimated blood loss, 3. peritoneal soiling, 4. presence of malignancy, and 5. mode of surgery, which could accurately reflect intraoperative condition and is considered reliable for surgical audits [22–23]. Yet, there were no differences in operative and physiological POSSUM score between groups, which indicates that there were diminished impacts on operative severity (Table 2).

Patient exposure to PPI therapy was reported to be associated with an increased occurrence of pneumonia [28–29] and an increased risk of community acquired pneumonia [30–31]. However, most studies focused on stable patients rather than critically ill patients. In contrast, a recent study showed that the observed association might be due to confounding factors depending on the indication of PPI therapy [32]. In the current series, there was no difference in intravenous PPI use between the two groups (Table 1).

The majority of pneumonia cases in the intensive care unit were mostly attributed to aspiration or ventilator complications due to oropharyngeal accumulated secretions with pathogens above the endotracheal tube cuff. Micro-aspirations of these subglottic secretions resulted because of an underinflated tracheal cuff [33]. Recent reports reveal that the continuous control of tracheal cuff pressure is associated with a significantly reduced occurrence of micro-aspirations of gastric contents in critically ill patients [34–35]. In this study, we applied strict VAP prevention bundle, as well as vigorous chest care and continuous control of tracheal cuff pressure to our patients. This practice may account for the similar occurrence rates of pneumonia between the two groups. Therefore, we assume that the risk of pneumonia might not be increased in critically ill PPU patients with nasogastric feeding provided that they receive an appropriate care bundle and program.

There was a longer length of stay in the FJ group compared to the NG group (Table 2). This could be attributed to the status that feeding jejunostomy procedures were performed. Therefore, there could be more complicated situations and procedures that resulted in a prolonged length of hospital stay (e.g. infected wound care, intraabdominal infection…etc.). However, there were no differences in severity scores, total ventilator days, infection rate, or survival rate between groups.

In addition, the FJ group was noted with significantly shorter intervals of establishment of enteral nutrition (Table 4), which could be due to eliminating the need to monitor residual gastric volumes, as was done in the NG group.

In summary, there were no significant differences in the occurrence of pneumonia between NG or FJ feeding routes in critically ill PPU patients who underwent surgery in this study. Our study is different from previous studies [13–16] because it used a single surgical group rather than mixed medical and surgical populations. Based on this result, we assume that similar treatment modalities can have varied outcomes when subgrouping the critically ill populations. Further studies are still needed to validate the results of this study.

### Limitations of the study

We recognize the limitations of this study, including its retrospective nature, small sample size and probable bias in case selection. These limitations may restrict our conclusions from the analysis. Additionally, because there were multi-factorial characteristics in patients with PPU and peritonitis who underwent surgery, it was difficult to collect all of the related data. Therefore, the evaluation of the physiological status and severity of these patients was done using the physiological scores rather than detailed clinical parameters. Though there were no differences in severity scores, the FJ group had a higher probability of abdominal turbid ascites/soiling or concern for postoperative leakage, which may have impacted the outcome. Another limitation is that the meticulous daily data collection was not always obtained in full and included. Yet, including these data could have resulted in a more thorough and precise analysis. Therefore, further multi-center randomized studies are warranted with predefined enrollment criteria for a better understanding of this issue.

## Conclusion

Feeding methods either through pre-pyloric or post-pyloric routes can be important for particular groups of patients. Our study indicates that there are no differences in the occurrence of pneumonia or mortality between the nasogastric and jejunostomy EN feeding routes in critically ill PPU patients who underwent surgery.

## Abbreviations

EN: enteral nutrition
PPU: perforated peptic ulcer
ESPEN: European Society for Parenteral and Enteral Nutrition
ASPEN: American Society for Parenteral and Enteral Nutrition
PPIs: proton pump inhibitors
APACHE II: Acute Physiology and Chronic Health Evaluation II
SOFA: Sequential Organ Failure Assessment
POSSUM: physiological and operative severity scores for the enumeration of mortality and morbidity
BMI: body mass index
VAP: ventilator associated pneumonia
ICU: intensive care unit
NG: nasogastric
FJ: (surgical) feeding jejunostomy
OR: odds ratio
CI: confidence interval
GCS: Glasgow coma scale
